# Soil Amendment with Poultry Manure, Biochar, and Coenzyme A Enhances Yield and Nutritional Composition of *Moringa oleifera* Lam.

**DOI:** 10.3390/foods14203527

**Published:** 2025-10-16

**Authors:** Baba Mamudu, Cristina García-Viguera, Diego A. Moreno, Eli Gaveh, Francis Appiah, Irene Idun, Sonia Medina, Raúl Domínguez-Perles

**Affiliations:** 1 Department of Horticulture, Kwame Nkrumah University of Science and Technology (KNUST), PMB, Kumasi P. O. Box 854, Ghana; mamudubaba1986@gmail.com (B.M.); eligaveh.canr@knust.edu.gh (E.G.); fappiahsp@gmail.com (F.A.); ireneidun@gmail.com (I.I.); 2 Laboratorio de Fitoquímica y Alimentos Saludables (LabFAS), CSIC, CEBAS, Campus Universitario de Espinardo, 25, 3100 Murcia, Spain; dmoreno@cebas.csic.es (D.A.M.); smescudero@cebas.csic.es (S.M.)

**Keywords:** *Moringa oleifera*, fertilisation, biomass production, glucosinolates, (poly)phenols, mineral content, trace elements

## Abstract

This study assessed the combined application of poultry manure (Pm), biochar (B), and coenzyme A (CoA) into soils to enhance *Moringa oleifera* Lam. growth, biomass yield, and nutritional and phytochemical composition. This approach allowed us to cover the gap of knowledge on sustainable, low-cost agronomic management alternatives suitable for smallholder systems. To achieve this objective a field experiment was conducted using three treatments (control (no amendment), Pm + B, and Pm + B + CoA) and four consecutive harvests were monitored. Morphological traits (height, stem diameter, number of branches, and leaf yield) were recorded, and phytochemical analyses of glucosinolates and (poly)phenols were performed via HPLC-DAD-ESI/MSn. Mineral and trace elements were quantified by ICP-OES. The main results retrieved allowed describing the capacity of the combined use of Pm + B + CoA to enhance plant growth and productivity, thus increasing the moringa trees’ height of 226.3 by 39.5%, on average, relative to control plants. ILeaf yield and branch number augmented up to 7.0-fold and 2.5-fold, respectively, under amendment treatments. Petiole girth also increased significantly by >50% (*p* < 0.01). Phytochemically, Pm + B + CoA significantly elevated total phenolics, vicenin-2, and quercetin acetyl-hexoside in leaves by 2.8-fold, on average, relative to control. The glucosinolate content also augmented as a result of the soil amendments assayed by 51.0%, on average, in stems and petioles, under Pm + B + CoA, compared to control samples. From these results, it can be concluded that the combined use of poultry manure, biochar, and CoA significantly improved *M. oleifera* growth, biomass yield, and nutritional quality, with a particular efficiency concerning (poly)phenolic accumulation. This low-cost, sustainable amendment strategy provides a viable agronomic solution in regions suffering socioeconomic constraints that hinder access to high-cost agronomic management options. Therefore, this approach effectively links ecological soil management with improved productivity, nutritional value, and potential for food industries.

## 1. Introduction

In recent years, interest in *Moringa oleifera* Lam. has increased due to its high nutritional and phytochemical value, which supports the use of its edible production and by-products in food, nutraceutical and cosmetic industries as valuable ingredients associated with health benefits [1], namely as moringa leaf powder, fortified foods, functional beverages, moringa seed oil, and functional extracts [2,3,4]. In close connection with these moringa-based products and their contribution to balanced nutrition, moringa cultivation has expanded worldwide, particularly in Africa, Latin America, the Caribbean, the Middle East, and Southern Europe [5]. Therefore, moringa crops are now exposed to augmented productive pressure since they suffer continuous harvesting. This agronomic demand can affect soil quality and local vegetation if not managed sustainably [6]. To date, there is limited information on the best management alternative to improve the production of moringa foliage in small acreages. Consequently, the development of sustainable soil management strategies to enhance moringa biomass yield and quality is of great interest [7].

The exploration of agronomical management factors involved in moringa productivity, such as soil fertility and nutrient availability, has allowed the identification of developing new sustainable soil amendments alternatives as a research priority. Enhancing these factors would contribute to optimising critical indicators of the moringa quality, such as biomass accumulation, secondary metabolism, and mineral composition.

To achieve this objective, the utility of biochar as an organic amendment has been demonstrated in its capacity to boost physicochemical properties of soils, nutrient cycling, contaminant adsorption, and microbial activity [8,9,10], thus improving soil ecological functions [8,11,12]. The achievable valuable functionality associated with the addition of biochar into soils can be complemented, for instance, by applying poultry manure, which supplies a range of nutrients essential for plant growth and development [13]. In addition to these amendment options, in recent years, coenzymes and related cofactors have been identified as promising bio-stimulants providing a valuable input on nutrient assimilation and stress tolerance in plants, resorting to fine-tuning enzymatic reactions associated [14]. However, to our knowledge, the effect of coenzyme applications has been scarcely characterised [15]. In addition, despite this cumulative evidence, most results available on the utility of the diverse soil conditioners have been based on the characterisation of individual conditioners [13,16], with limited information available on their interactive or synergistic impacts on moringa crop performance. This fact limits potential agronomic advantages concerning plant growth and secondary metabolism, especially concerning *M. oleifera*. Therefore, nowadays, there is a need for additional research on the combined application of soil amendments on moringa cultivation that is expected to synergistically improve soil fertility, nutrient availability, and plant physiological responses, potentially leading to greater increases in moringa leaf production, yield, and quality compared to individual treatments [17]. This study would contribute to optimising sustainable soil management practices and enhancing the moringa supply and value chains.

Based on these antecedents, the present study unravels the effect of soil-combined amendments (poultry manure, biochar, and coenzyme A (CoA)) on the moringa biomass yield (growth and harvest yield) and quality of moringa tissues regarding phytochemical (glucosinolates and phenolics compounds) and mineral (nutrients (phosphorus (*P*), calcium (*Ca*), magnesium (*Mg*), potassium (*K*), sodium (*Na*), and sulphur (*S*)) and trace elements (copper (*Cu*), iron (*Fe*), manganese (*Mn*), molybdenum (*Mo*), and zinc (*Zn*))) composition, thus shedding light on the advance provided on soil amendments by the combined use of conditioners.

## 2. Materials and Methods

### 2.1. Chemicals and Reagents

The standards sinigrin (sinigrin monohydrate from *Sinapis nigra*) and quercetin 3-*O*-glucoside were purchased from Phytoplan Diehm & Neuberger (Gmbh, Heidelberg, Germany) and Sigma-Aldrich (Steinheim, Germany), respectively. Acetic and hydrochloric acids were from Panreac Labs (Barcelona, Spain). All LC-MS-grade solvents (methanol, acetonitrile, and deionised water) were supplied by J.T. Baker (Philipsburg, NJ, USA).

### 2.2. Experimental Design, Climate, and Treatments

The growing season (2023) started on 22 February. The experimental moringa (*M. oleifera*) crop was conducted as a comparative trial under a Randomised Complete Block design (RCBD), including two experimental treatments (poultry manure and biochar (Pm + B)) and poultry manure, biochar, and CoA (Pm + B + CoA). The planting space was 30 × 30 cm, with certified seeds sown at a depth of 2–3 cm. Following germination, 8–10 days after sowing, the moringa seeds grew uninterrupted, responding to the initial treatment application. Sixty (60) centimetres was established between plots. Three experimental conditions were established: first, control conditions without any additional amendments; second, the application of Pm + B; and third, the application of Pm + B + CoA.

The field trial was carried out at the Experimental Field of the Kwame Nkrumah University of Science and Technology, Kumasi, Ghana (0°26′ N, 0°6′ E) during the 2023 season (February–November 2023). There was a bimodal precipitation pattern, with approximately 55% of the annual rainfall occurring between February and July and 30% between September and November. The average temperature recorded ranged between 27 °C and 29 °C, with the hottest months being February, March, and April.

The soil was prepared through mechanical ploughing and harrowing. Garden lines and pegs were used to demarcate the plots after ploughing. An 80 cm contour hedge was established around the site to protect the experimental area. The field consisted of one block comprising nine plots of 1.2 × 1.2 m (the three treatments (control, Pm + B, and Pm + B + CoA) replicated three times). Handheld tools (rake and hoe) were employed to achieve a level surface, thus breaking up soil lumps and ensuring uniformity. For each experimental plot, according to the specific conditions, the soil amendments were applied at the following rate, 8 kg/plot (56 tons/ha) of poultry manure and 4 kg/plot (28 tons/ha) of Biochar, following previous descriptions in the literature [18,19]. Since no previous assessment of CoA application in field treatments has been described in the literature, the concentration applied (400 mg per L, then applying 5 L/plot) was adjusted according to pilot data gathered in the geographical area upon testing from 0 to 10 L/plot. The amendments were applied 90 days after planting with intervals of 60 days between harvests. Furrow and sprinkler irrigation maintained optimal soil moisture for germination and seedling growth. The weeds were controlled by hand and hoeing between beds and rows.

For each experimental treatment, measurements were taken from three plots at four consecutive harvests, with a 60-day interval between each harvest. For productive parameters, 5 samples were analysed in each plot, treatment, and harvest considered that gave rise to 15 replicates per treatment (control, Pm + B, and Pm + B + CoA) to evaluate plant height (in cm), the number of pinnae and branches, stem girth, and fresh and dry weight, which were expressed per experimental tree.

In addition, for phytochemical and mineral analyses, all harvests corresponding to the separate treatments were considered together, aiming to obtain practical information on the total production content obtained in a single season. Thus, the 20 samples per tissue (5 samples × 4 harvests) obtained from each of the three plots per treatment were thoroughly mixed to form three composite samples per plot, yielding 9 replicates per treatment (*n* = 9). The samples, once oven-dried, were ground into a fine powder and stored at −20 °C for further analysis. Samples were transported to the laboratory in an open basket, and the three distinct parts (leaves, stem, and petiole) were separated by hand.

### 2.3. Extraction and Determination of Intact Glucosinolates and Phenolic Compounds

Dried powder (100 mg) was extracted in 1.5 mL of methanol/deionised water (70:30, *v*/*v*) for 20 min at 70 °C, vortexed every 5 min to improve extraction, and then centrifuged at 20,000× *g*, 10 min, at 4 °C (Sigma 1–13, B. Braun Biotech Intl., Osterode, Germany). The supernatants were collected and filtered through a 0.22-μm inorganic membrane filter (ANOTOP 10 plus, Whatman, Maidstone, UK).

HPLC-DAD-ESI/MSn analyses were developed using an Agilent HPLC 1100 equipped with a photodiode array detector (model G1315B) and a mass spectrometer in series (Agilent Technologies, Waldbronn, Germany). The HPLC system consisted of a binary pump (model G1312A), an autosampler (model G1313A), a degasser (model G1322B), and a sample cooler (model G1330B). The instrument was controlled by ChemStation software (v. 08.03). The separate intact glucosinolates and phenolic compounds were identified from the extracted samples according to the retention time, the parent mass, and fragmentation patterns (*m*/*z* [M−H] and MS2 [M−H]) in the HPLC-DAD-ESI-MSn, carried out on a C18 Kinetex column (150 × 4.6 mm, 5 μm particle size; Phenomenex, Macclesfield, UK). Water/formic acid (99:1, *v*/*v*) and acetonitrile were used as mobile phases A and B, respectively, with a flow rate of 0.8 mL/min. The linear gradient started with 1% solvent B, reaching 17% B at 17 min, 25% B at 26 min, and 60% B at 42 min. The injection volume was 20 μL. Spectral data from all peaks were detected in the 200–600 nm range, and chromatograms were recorded at 227 and 330 nm for glucosinolates and phenolic compounds, respectively. The mass detector was an ion trap spectrophotometer (Model G2445A) equipped with an electrospray ionisation interface (Agilent Technologies, Waldbronn, Germany) and wastrolled by LCMSD software (version 4.1—Agilent Technologies, Waldbronn, Germany). Ionisation conditions were adjusted at 350 °C and 4 kV for capillary temperature and voltage, respectively. The nebuliser pressure and flow rate of nitrogen were 60.0 psi and 11 L/min, respectively. The full-scan mass covered the range from *m*/*z* 10 to 1200. Collision-induced fragmentation experiments were performed in the ion trap using helium as the collision gas with voltage ramping cycles from 0.3 to 2.0 V. Mass spectrometry data were acquired in the negative ionisation mode. The concentration of glucosinolates was obtained by semi-quantitative determination using sinigrin as an external standard (227 nm) (recovery: ≥85.0%; linearity: 1.000-0.016 µmol/mL; limit of detection (LOD): 0.008 µmol/mL, and limit of quantification (LOQ): 0.016 µmol/mL), because of the similar structure to the glucosinolates in the sample, while phenolics were semi-quantified as equivalents of quercetin 3-*O*-glucoside (360 nm) (recovery: ≥85.0%; linearity: 1.000-0.004 µmol/mL; LOD: 0.001 µmol/mL, and LOQ: 0.004 µmol/mL). Standard curves were freshly prepared each day of analysis.

### 2.4. Analysis of Mineral Nutrients and Trace Elements

The analysis of *P*, *Ca*, *Mg*, *K*, *Na*, *S*, *Cu*, *Fe*, *Mn*, *Mo*, and *Zn* was carried out after HNO_3_–HClO_4_ (2:1) acid digestion of the dried plant material, was performed by ICP-spectrometry (OES Thermo ICAP 6000 SERIES^®^; Thermo Electron Corp., Franklin, MA, USA), diluting the extract aliquot with LaCl_3_ + CsCl, according to Domínguez-Perles et al. [20].

### 2.5. Statistical Analysis

Results are presented as means ± standard deviation (SD). The normality of the results distribution and the homogeneity of variance were assessed by Kolmogorov–Smirnov and Levene tests, respectively. Due to the normal distribution of data, a one-way analysis of variance (ANOVA) was applied to compare the experimental conditions. Tukey’s multiple range test was developed when the ANOVA test displayed significant differences by SPSS 29.0 (LEAD Technologies, Inc., Chicago, IL, USA). The level of statistical significance was set at *p* < 0.05.

## 3. Results

### 3.1. Plant Height of Moringa oleifera as Influenced by Soil Amendments

**Figure 1:**
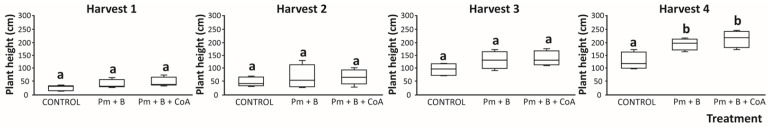
*Moringa oleifera* L. height (cm) achieved in the four consecutive harvests during growth under different treatments (control, poultry manure + biochar (Pm + B), and poultry manure + biochar + coenzyme A (Pm + B + CoA)). Different lowercase letters within each separate blot indicate values significantly different at *p* < 0.01 according to one-way analyses of variance (ANOVA) and Tukey’s multiple range test (*n* = 15).

When analysing the moringa tree growth, the joint application of poultry manure, biochar, and CoA (Pm + B + CoA) provided maximum plant height of up to 226.3 cm, on average, which was statistically different (*p* < 0.05) from the values obtained in trees developed under control conditions (no soil amendments) (Figure 1). Thus, concerning the growth experienced throughout four consecutive harvests, no significant difference in the *M. oleifera* plant height was observed in the first three harvests. Further, in the fourth harvest, the results demonstrated the relevance of applying poultry manure and biochar, with or without CoA (Pm + B or Pm + B + CoA, respectively), as a significant difference was observed relative to the control (Figure 1).

These results agree with previous descriptions of the capacity of organic manures to supply required plant nutrients after mineralisation in soil. In this regard, organic manure contributes to the formation of soil aggregates and stability. Specifically, enhanced porosity and water holding capacity of the soil might also have a favourable influence to support the root system through enhanced nutrient flow that, in turn, increases the growth and yield of biomass [21]. The amount and kind of added biochar affected the crop’s increased yield. Different biochars have distinct effects on specific crops [22]. Biochar enhances soil carbon, improving water retention and nutrient availability. However, its effects on agricultural productivity are variable and context-dependent. Potential drawbacks reported in the literature include nutrient imbalances, changes in soil pH and electrical conductivity, impacts on soil microbial activity, and the possible release of toxicants [23]. Therefore, although biochar can provide soil benefits, its use in agriculture should be carefully managed. The cofactor CoA participates in the tricarboxylic acid cycle, nutrient oxidation, histone acetylation and synthesis of lipids and glycans [24]. On this basis, CoA, which interacts with the soil and plants, was introduced to aid the availability of nutrients to the moringa plants during the experimental process.

### 3.2. Number of Pinnae of Moringa oleifera as Influenced by Soil Amendments

**Figure 2:**
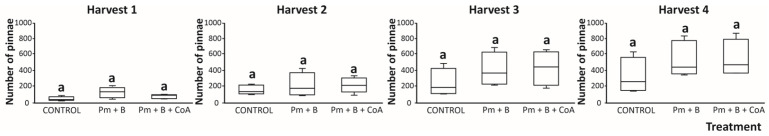
Number of pinnae of *Moringa oleifera* L. developed in the four consecutive harvests during growth under different treatments (control, poultry manure + biochar (Pm + B), and poultry manure + biochar + coenzyme A (Pm + B + CoA)). Different lowercase letters within each separate blot indicate values significantly different at *p* < 0.01 according to one-way analyses of variance (ANOVA) and Tukey’s multiple range test (*n* = 15).

After rigorous examination of the pinnae of *M. oleifera* trees during four consecutive harvests, in the first one, no significant difference in the number of pinnae (52 pinnae) were recorded, on average, by all field experiment conditions (control, Pm + B, and Pm + B + CoA)). Similar results were observed in the second harvest, as no statistical difference was found in the number of pinnae developed by the moringa trees (Figure 2). In the third and fourth harvests, the application of Pm + B and Pm + B + CoA demonstrated better performance relative to the control. However, the slight advantage observed when applying Pm + B + CoA was not statistically different (*p* > 0.05) (Figure 2).

Moringa leaves are characterised by being alternate, compound leaves with multiple layers of pinnae. Moringa leaves are compound, meaning they are made up of smaller leaflets attached to a central stalk called the rachis. They are often described as two or three times pinnate, meaning they have pinnae branching out from the main rachis, which themselves bear smaller leaflets. The pinnae are the structures that branch out from the main leaf stalk. They are typically opposite and spaced along the rachis. Each pinna bears multiple leaflets. The individual leaflets are generally dark green, elliptical to obovate, and about 1–2 cm in length. They are often described as 75–150 leaflets per leaf [25]. According to these results, the main outcomes gathered throughout the four consecutive harvests suggest a growing trend that aligns well with variation attributable to the Pm + B + CoA treatment. This is evident in the control, which exhibited lower pinnae during the entire growth period monitored, particularly in harvests 3 and 4. Additionally, the application of Pm + B Pm + B + CoA enhanced the pinnae formation of the moringa plant, although no significant difference was observed in any harvest.

### 3.3. Petiole Girth of Moringa oleifera Tree as Influenced by Soil Amendments

**Figure 3:**
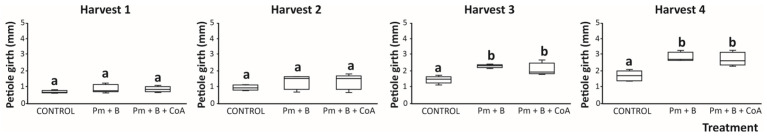
Petiole girth (mm) of *Moringa oleifera* L. developed in the four consecutive harvests during growth under different treatments (control, poultry manure + biochar (Pm + B), and poultry manure + biochar + coenzyme A (Pm + B + CoA)). Different lowercase letters within each separate blot indicate values significantly different at *p* < 0.01 according to one-way analyses of variance (ANOVA) and Tukey’s multiple range test (*n* = 15).

Petiole girth refers to the thickness or diameter of the petiole and is a basic parameter that helps to understand the growth and development of the moringa tree. In this regard, the present article monitored the effect of the different soil amendment alternatives assayed on the petiole diameter (Figure 3). In this regard, the application of poultry manure and biochar, with and without CoA provided no significant variation (*p* < 0.05) in the petiole in the first two harvests (0.85 and 1.26 mm, respectively).

On the contrary, statistical significance was observed concerning stem girth during the third and fourth harvests of growth, which evidenced the capacity of applying Pm + B to enhance this development parameter that provided 52.5% and 61.4% higher petiole girths, respectively, when compared with controls (Figure 3). Interestingly, no effect of including the CoA in the soil amendments assayed was recorded (Figure 3).

### 3.4. Number of Branches of Moringa oleifera as Influenced by Soil Amendments

**Figure 4:**
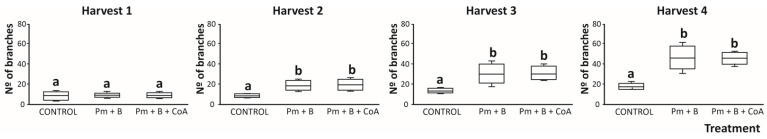
Number of branches of *Moringa oleifera* L. obtained in four consecutive harvests during growth under different treatments (control, poultry manure + biochar (PM + B), and poultry manure + biochar + coenzyme A (Pm + B + CoA)). Different lowercase letters within each separate blot indicate values significantly different at *p* < 0.01 according to one-way analyses of variance (ANOVA) and Tukey’s multiple range test (*n* = 15).

The number of branches of the moringa tree is an important parameter informing about the optimal development and growth of the moringa plant [26]. In this regard, most findings on the number of branches of *M. oleifera* in this study suggested a significant variation resulting from the different soil amendments assayed and throughout the four consecutive harvests. The number of branches produced during the first producing period did not differ significantly between soil treatments (Figure 4). Alternatively, in the second to fourth harvests, there was a significant difference in the number of branches produced under soil amendments treatments (Pm + B and Pm + B + CoA) relative to control as Pm + B and Pm + B + CoA treatments that provided the highest value (22, 31, and 47 branches, on average, for the second, third, and fourth harvests, respectively, Figure 4) in comparison with the untreated control (9, 14, and 19 branches, on average, correspondingly). These results demonstrated the efficiency of applying soil amendments.

The branches of the moringa tree are delicate and open, often drooping. They can be pruned to encourage a more compact growth habit, which can improve the yield of leaves and pods. Moringa leaves and branches have been used in traditional medicine to treat various ailments, including inflammation, pain, and infections and, therefore, constitute a valuable [27].

### 3.5. Leaf, Stems, and Petioles’ Yield of Moringa oleifera as Influenced by Soil Amendments: Fresh and Dry Matter Assessments

#### 3.5.1. Fresh and Dried Leaves Yield

**Figure 5:**
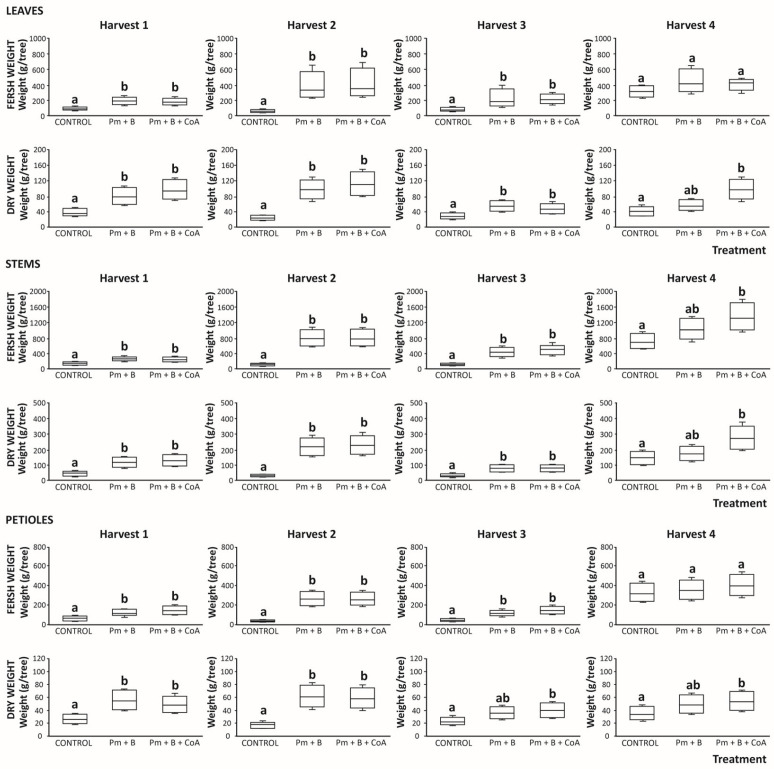
Fresh and dry weight (g) of leaves (A), stems (B), and petioles (C) of *Moringa oleifera* L. produced in the four consecutive harvests of growth under different treatments (control, poultry manure + biochar (Pm + B), and poultry manure + biochar + coenzyme A (Pm + B + CoA)). Different lowercase letters within each separate blot letters indicate values significantly different at *p* < 0.01 according to one-way analyses of variance (ANOVA) and Tukey’s multiple range test (*n* = 15).

When monitoring the plant material produced throughout the four harvests assessed, the fresh leaves’ weight recorded ranged between 90 and 439 g fresh weight (fw)/tree (Figure 5). The plant material produced evolved throughout four consecutive harvests. Thus, in the first harvest, the soil amendments assayed (Pm + B and Pm + B + CoA) produced an average amount of plant material of 189 g fw per tree, which was almost 2.0-fold higher than the production recorded under control conditions (Figure 5). This difference was even higher in the second harvest, when the soil management provided >7.0-fold plant material in comparison with control conditions. In the third and fourth harvests, the differences were reduced to 2.7 and 1.4-fold, respectively (Figure 5), thus demonstrating the assessed alternatives of soil amendments as valuable forms for enhancing moringa tree production in all cases.

Similar behaviour was observed concerning dried material. Again, in this aspect, the soil amendments improved the production capacity of moringa trees during all weeks and for almost all treatments. The advantages of applying poultry manure and biochar (with and without CoA) increased during the first two growing periods (harvests 1 and 2) from 2.4 to 4.4-fold. Thus, concerning this parameter, no significant differences were recorded between treatments (Figure 5). These differences were reduced up to 1.6 times, on average (not significantly at *p* > 0.05) during tree growth before the third and fourth harvests in soils amended by applying Pm + B. Interestingly, when the soil amendment included the use of CoA, the advantage over the control conditions was 2.1-fold, on average (Figure 5).

#### 3.5.2. Fresh and Dried Stems Yield

Concerning stems, which constitute an indicator of the growth of the moringa plant [17], when monitoring the amount produced throughout the four growing periods before harvesting, the fresh stems’ weight recorded ranged between 85 and 1780 g fw/tree (Figure 5). The stems in the first harvest from trees grown upon soil amendments (Pm + B and Pm + B + CoA) constituted an average amount of plant material of 258 g fw/tree, which was almost 1.8-fold higher than the production recorded under control conditions (Figure 5). As previously mentioned regarding the leaves, this difference was even higher in the second harvest, when the soil management provided 8.2-fold stem material in comparison with control conditions. The differences recorded in the first two harvests were reduced to 3.6 and 1.7-fold, respectively, in the third (*p* < 0.01) and fourth (*p* > 0.01) harvests, respectively, concerning control samples (Figure 5), evidencing the advantage of amending soils to enhance the moringa production, at least, during the first two harvests (120 days).

As expected, the soil amendments improved the production capacity of the moringa tree in all harvests and for almost all treatments for dried materials. The advantages of applying poultry manure and biochar (regardless of the cofactors addition) increased the production of dried stems from 2.3 times to 7.4-fold, in the first three harvests (Figure 5). Again, concerning stems, no significant differences were recorded between treatments in this period (Figure 5). These differences were reduced up to 1.2-fold, on average (no significant, *p* > 0.05), in the fourth harvest for tree growth in soils amended by applying poultry manure with biochar. Interestingly, when the soil amendment included cofactors, the advantage over the control conditions was statistically significant (by a 1.9-fold increase) (Figure 5).

#### 3.5.3. Fresh and Dried Petioles Yield

Regarding petioles, associated with moringa production, when monitoring the amount produced throughout the harvest period, the fresh petiole weight recorded ranged between 29 and 550 g fw/tree (Figure 5). The petiole matter produced in first harvest by trees grown upon soil amendments (Pm + B and Pm + B + CoA) produced an average amount of plant material of 141 g fw/tree, which was almost 1.9-fold higher than the production recorded under control conditions (Figure 5). As referred to concerning leaves and stems, this difference was even higher in the second harvest, when the soil management provided 6.6-fold stem material in comparison with control conditions. The differences observed in the first two harvests were reduced in the third and fourth harvests, when the average augmentation obtained by applying soil amendments relative to the control conditions was 2.7 and 1.2-fold, respectively. Thus, the treatments in the third week were the only significantly different (Figure 5). The timing of planting and other management practices caused a significant effect on the growth and survival of moringa trees.

Optimal timing for planting, particularly in the dry season, includes irrigation for the first two months to establish the root systems, after which moringa trees become more resistant to drought. Spacing also plays a role, with narrower spacing generally leading to better survival rates in the initial months after planting. Additionally, temperature and climate conditions influence moringa growth, with optimal temperatures between 25 and 35 °C promoting growth and higher pod and leaf production [28].

As expected, the soil amendments improved the production capacity of moringa trees across all weeks and for nearly all treatments involving dried materials. The advantages of applying poultry manure and biochar (independently of the use of enzymatic cofactors) increased the production of dried stems from 1.5 to 3.4-fold, during the 4 harvests (Figure 5). Concerning petioles, no significant differences were recorded between Pm + B and control in the third and fourth harvests (*p* > 0.05, Figure 5). Alternatively, the application of Pm + B + CoA allowed maintaining significant differences (*p* < 0.01) in the last two harvests, which were quantified as 1.8 (harvest 3) and 1.5 (harvest four)-fold higher than the control (Figure 5). The application of manure and biochar could have aided in the rapid cell division particularly mesophyll cells which also contain chloroplast. Comparable patterns have been previously described in the readings, where chlorophyll content in the leaves, when using soil fertilisers, was greater in the third and fourth harvest plants [29,30]. These authors reported that mesophyll cells play a crucial role in the photosynthetic process of leaves. Further study showed that the application of charcoal dust to peanuts increased leaf photosynthetic rate and the maximum electron transport rate [31]. The presence of sunlight, together with available soil nutrients, facilitated leaf yield due to the essential role of light intensity in determining productivity [32]. A study by Dalirie et al. (2010) reported that an increase in shading decreases leaf area, which consequently reduces dry matter production and vice versa. This entails a reduction in photosynthetic processes [33]. Furthermore, under no shading, sunlight is readily available for photosynthetic processes, leading to the complete plant growth phases (vegetative, reproductive, and dry matter partitioning). The increase in Pm + B dust soil amendments has also played a significant role in the increase in leaf yield, as it helps the plant to manufacture more food under favourable environmental conditions. This is in harmony with Kilic et al., who revealed that an increase in yield is firmly associated with the plants’ ability to produce more biomass in the presence of favourable conditions such as sunlight, nutrients, and water [34].

### 3.6. Glucosinolate Content of the Tissues with the Soil Amendments

Moringa plant tissues were further evaluated for their interest as a source of bioactive phytochemicals, especially glucosinolates and phenolic compounds. In this context, when monitoring the effect of soil amendments on the glucosinolates content, the different tissues under study (petiole, leaf, and stem) revealed the presence of two main glucosinolates (4-(α-L-rhamnopyranosyloxy)benzyl-glucosinolate and 4′-*O*-acetyl-4-(α-L-rhamnopyranosyloxy)benzyl-glucosinolate), which were identified according to their retention time, parent ion, and specific fragmentation pattern, in comparison with previous reports in the literature [35]. Thus, 4-(α-L-rhamnopyranosyloxy)benzyl-glucosinolate and 4′-*O*-acetyl-4-(α-L-rhamnopyranosyloxy)benzyl-glucosinolate exhibited parent ions at *m*/*z* [M-H] 570 and 612 arbitrary mass units (amu), both of them featured by a similar structure except for the presence of an acetyl group, located at C2′, C3′, and C-4′ on the α-L-rhamnopyranosyl unit. The identity of the revealed glucosinolates was further confirmed by comparing the MS2 spectra corresponding to each compound with those reported in the literature. Thus, the most characteristic fragments considered for confirmation purposes were *m*/*z* MS2[M-H] 97 and 259 amu, respectively [35].

**Figure 6:**
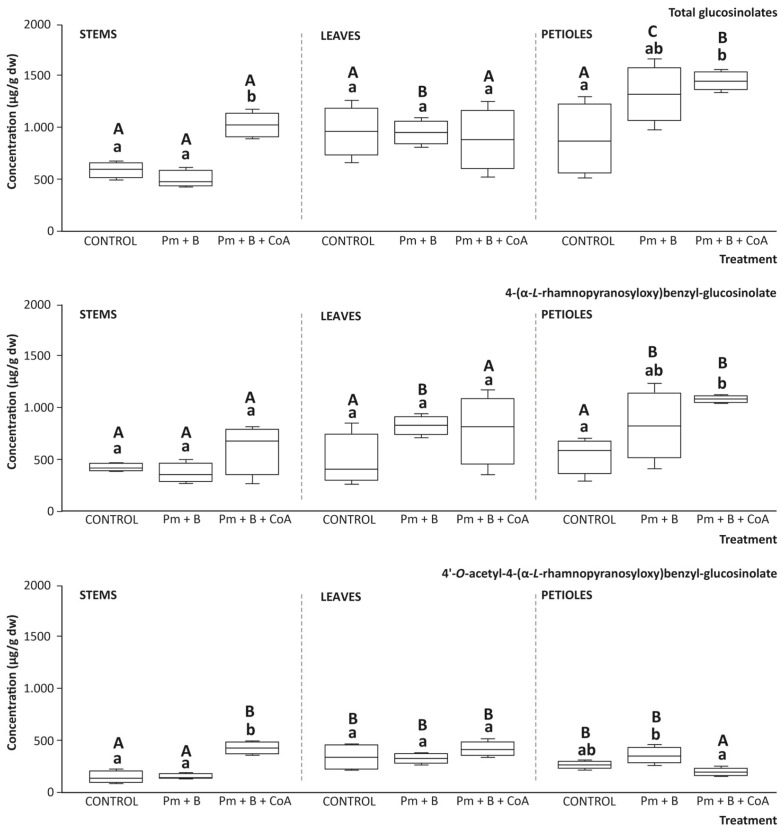
Content of total glucosinolates, 4-(α-*L*-rhamnopyranosyloxy)benzyl-glucosinolate, and 4′-*O*-acetyl-4-(α-*L*-rhamnopyranosyloxy)benzyl-glucosinolate (μg/g dw) in *Moringa oleifera* L. tissues (stems, leaves, and petioles). Boxes with distinct lowercase letters within each separate blot are significantly different between treatments (control, poultry manure + biochar (Pm + B), and poultry manure + biochar + coenzyme A (Pm + B + CoA)), and distinct capital letters are significantly different between tissues, at *p* < 0.05, according to one-way analysis of variance (ANOVA) and Tukey’s multiple range test (*n* = 9).

The quantitative glucosinolate profile of leaves, stems, and petioles obtained under control conditions and resulting from the application of Pm + B and Pm + B + CoA revealed matching profiles and similar concentrations, with minor differences between tissues. These results indicated the highest concentrations of 4-(α-L-rhamnopyranosyloxy)benzyl-glucosinolate in petioles, with average values of 544.00 μg/g dw, 830.00 μg/g dw, and 1086.23 μg/g for control, Pm + B, and Pm + B + CoA, respectively. Thus, petioles surpassed the levels recorded in leaves by 13.5%, 0.7%, and 27.9%, respectively, and in stems by 22.2%, 55.7%, and 44.1%, correspondingly (Figure 6).

Concerning 4′-*O*-acetyl-4-(α-L-rhamnopyranosyloxy)benzyl-glucosinolate, in trees under control conditions, the highest concentration corresponded to leaves (345.25 μg/g), which surpassed the amount in stems and petioles by 62.3% and 21.2%, respectively (Figure 6). Interestingly, when applying the soil amendments assessed, the concentrations augmented in the different tissues, in close association with the specific treatment. After adding Pm + B into soils, the concentration of 4′-*O*-acetyl-4-(α-L-rhamnopyranosyloxy)benzyl-glucosinolate remained almost unaltered in stems and leaves, while in petioles, it rose by 17.3%. Alternatively, the application of Pm + B + CoA provided values 69.0% and 22.4% higher than the control in leaves and stems, respectively (Figure 6). Thus, when comparing moringa tissues, concerning total glucosinolates and 4-(α-L-rhamnopyranosyloxy)benzyl-glucosinolate, the highest content was recorded in petioles. Interestingly, the noted differences were strongly influenced by the application of soil amendments, especially by applying Pm + B + CoA (Figure 6). The application of Pm + B + CoA was the most efficient intervention since significantly augmented the concentration of total glucosinolates and 4-(α-L-rhamnopyranosyloxy)benzyl-glucosinolate in leaves and petioles. Alternatively, the Pm + B-based treatment was more efficient in modulating the concentration of these two parameters in petioles (Figure 6). This finding evidenced the different physiological functions of the separate tissues analysed and, thus, the achievement of specific concentrations and the distinct effect of management interventions on the glucosinolate content [36].

Indeed, the effect of soil amendments on the glucosinolates profile of the moringa tissues assessed is in good agreement with the metabolic origin and physiological role of these compounds. In this regard, environmental factors and cultural practices significantly influence the secondary metabolites in plants, including glucosinolates. However, this effect is closely related to the species under consideration, in the present work, *M. oleifera*. Thus, previous research on the association between fertilisation and glucosinolate metabolism in this crop, under similar agro-climatic conditions in Ghana, provided evidence that fertilisation can modulate the quantitative glucosinolate profile [35]. Hence, although this study reported harvest time as a relevant factor influencing glucosinolate metabolism and accumulation in tissues, this was not further confirmed in the present work; a cushioning effect of the soil amendments applied could be hypothesised. Therefore, the main results obtained on the glucosinolate content of leaves, stems, and petioles of moringa upon successive harvests suggest that selecting appropriate cultivation practices would contribute to optimising the production of enhanced health-promoting moringa plants based on the content of these bioactive organosulfur compounds [35]. In this regard, variations in the glucosinolate profile have been associated with soil fertilisation practices (e.g., poultry manure and biochar) that enhance the availability of *S* and *N*, hence boosting glucosinolate metabolism and concentration in plants [36]. In addition, the effect of fertilisers is reinforced by the joint application of cofactors, which provides the highest glucosinolate concentration. Enzymes and cofactors in soil play key functions by catalysing biochemical processes to convert soil organic matter to available nutrients, while simultaneously maintaining the health, stability, and resilience of ecosystems [37].

Additionally, the similar profiles in the separate moringa tissues led to envisioning joint uses for all of them, thus contributing to simplifying the crop management and plant material processing. Anyhow, the resulting description of the glucosinolate profile of moringa leaves, stems, and petioles will help to identify their potential for generating new ingredients with added value and a significant impact on agronomic sustainability and the value as a source of bioactive phytochemicals.

### 3.7. Phenolic Content of the Tissues with the Soil Amendments

Beyond glucosinolates, the effect of soil amendments on the phytochemical burden of stems, leaves, and petioles of moringa was further analysed by determining the content of (poly)phenolic compounds. The different tissues assessed displayed the presence of two phenolic compounds (apigenin di-*C*-glucoside (vicenin-2) and quercetin acetyl-hexoside), which were found in concentrations higher than the limit of detection and quantification (LOD and LOQ, respectively), as the lowest concentration that can be detected and quantified, respectively, as presenting signal to noise (S/N) ratios higher than 3:1 and 10:1, correspondingly [37]. As referred to for glucosinolates, individual phenolic compounds were identified according to their retention time, parent ion, and specific fragmentation pattern, in comparison with previous reports in the literature [38]. Thus, apigenin di-glucoside (vicenin-2) and quercetin acetyl-hexoside exhibited parent ions at *m*/*z* [M-H] 593 and 505 amu, respectively. The fragmentation of both compounds rendered an MS2 spectrum, including the most characteristic fragments described in the literature, considered for confirmation purposes (*m*/*z* MS2[M-H] 353 and 301 amu, correspondingly) [38].

**Figure 7:**
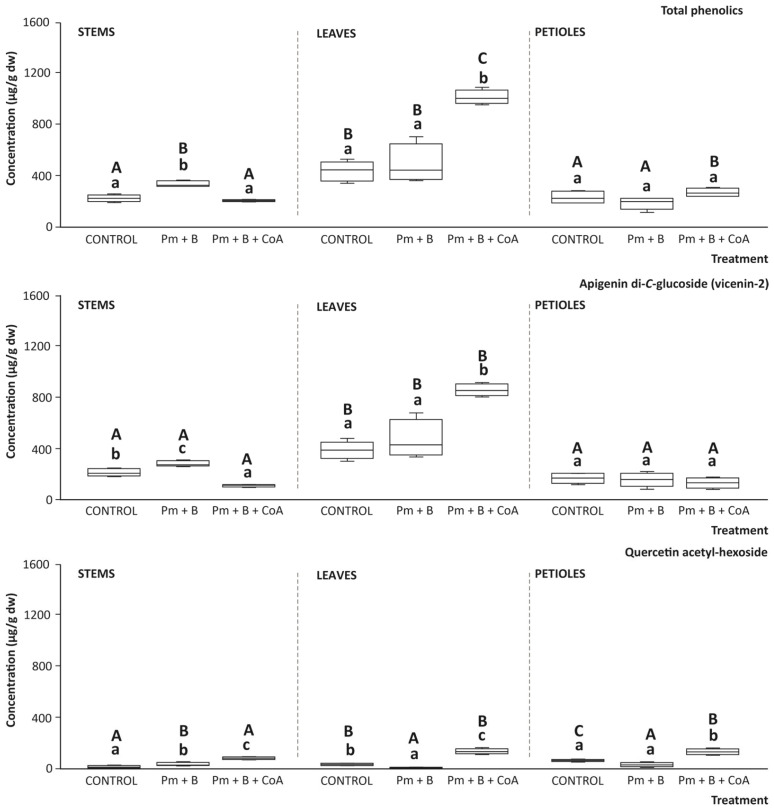
Content of total phenolics, apigenin di-*C*-glucoside (vicenin-2), and quercetin acetyl-hexoside (μg/g dw) in *Moringa oleifera* L. tissues (stems, leaves, and petioles). Boxes with distinct lowercase letters within each separate plot are significantly different between treatments (control, poultry manure + biochar (Pm + B), and poultry manure + biochar + coenzyme A (Pm + B + CoA)), and distinct capital letters are significantly different between tissues, at *p* < 0.05, according to one-way analysis of variance (ANOVA) and Tukey’s multiple range test (*n* = 9).

When assessing the quantitative phenolic profile, the main results obtained highlighted leaves as the best source of phenolic compounds (648.00 μg/g dw), surpassing stems and petioles by 51.8%, on average. Indeed, the latter tissues remained at a similar lower level. This trend was consistent in all the growing conditions assayed (Figure 7). The evaluation of the soil management treatments (Pm + B and Pm + B + CoA) on the capacity to modulate the total phenolic concentration showed a limited impact, except for leaves, where the latter treatment induced a significant augment of the concentration of both total and individual phenolics (Figure 7).

Concerning the contribution of the individual phenolics identified, the highest amount was provided by vicenin-2, which exhibited a matching trend in comparison with total phenolics. Hence, as described for total phenolics, leaves displayed the highest concentrations of vicenin-2, which ranged between 401.25 and 872.50 μg/g dw, surpassing the average values recorded in stems and petioles, for all treatments, by 63.7% and 72.6%, respectively. This phenolic experienced an increasing concentration as a result of the soil amendments assayed. However, the increased concentration was only statistically significant when applying Pm + B + CoA (Figure 7). Alternatively, the assessment of moringa leaves, stems, and petioles on the quercetin acetyl-hexoside content evidenced minor (although significant) differences between tissues, according to its lower concentration relative to vicenin-2. Anyhow, the application of Pm + B + CoA was the treatment that increased in the most significant from the concentration of quercetin acetyl-hexoside in all tissues up to 90.25, 143.00, and 131.00 μg/g dw, for stems, leaves, and petioles, respectively, which surpassed the content achieved under control conditions or when applying soil amendments based on Pm + B by 72.2%, on average (Figure 7).

The distribution of phenolics among different tissues (leaves > petioles ≈ stems) would reflect tissue-specific metabolic activity and functional specialisation. Leaves, as primary photosynthetic organs, are often more exposed to environmental stressors (e.g., UV light), which are known to upregulate phenylpropanoid pathway enzymes [39]. This physiological role aligns with the higher phenolic content recorded in leaf tissues and supports their preferential use in nutraceutical applications.

The phenolic compounds in moringa are represented by apigenin di-*C*-glucoside and quercetin acetyl-hexoside. Among the soil treatments evaluated, the combined application of poultry manure, biochar, and cofactor significantly enhanced the total phenolic content, especially in leaves, suggesting a synergistic effect of organic and enzymatic amendments on phenolic metabolism [40], which was demonstrated, to our knowledge, for the first time in moringa. This fact is in agreement with the molecular functions of (poly)phenols, which are recognised antioxidants, and their accumulation in plant tissues is influenced by environmental conditions and agronomic practices [41]. The increase in vicenin-2 and quercetin derivatives under the Pm + B + CoA treatment could be associated with improved nutrient availability and root-soil interactions facilitated by the enzymatic activity [42]. In this regard, previous studies have reported that organic amendments, namely compost or biochar, can stimulate the biosynthesis of phenolics through improved soil fertility, microbial activity, and hormonal signalling in plants [43]. Thus, the addition of enzymes and cofactors to the soil amendment regime appears to amplify the biosynthesis of secondary metabolites, likely by accelerating nutrient mineralisation and enhancing plant nutrient uptake, which are critical for phenolic biosynthesis [44].

Thus, according to the main results, in practical terms, the use of poultry manure, biochar, and CoA as a combined soil amendment strategy not only improves plant growth and yield but also enhances the phytochemical richness of *M. oleifera*.

### 3.8. Mineral Nutrients and Trace Elements

**Figure 8:**
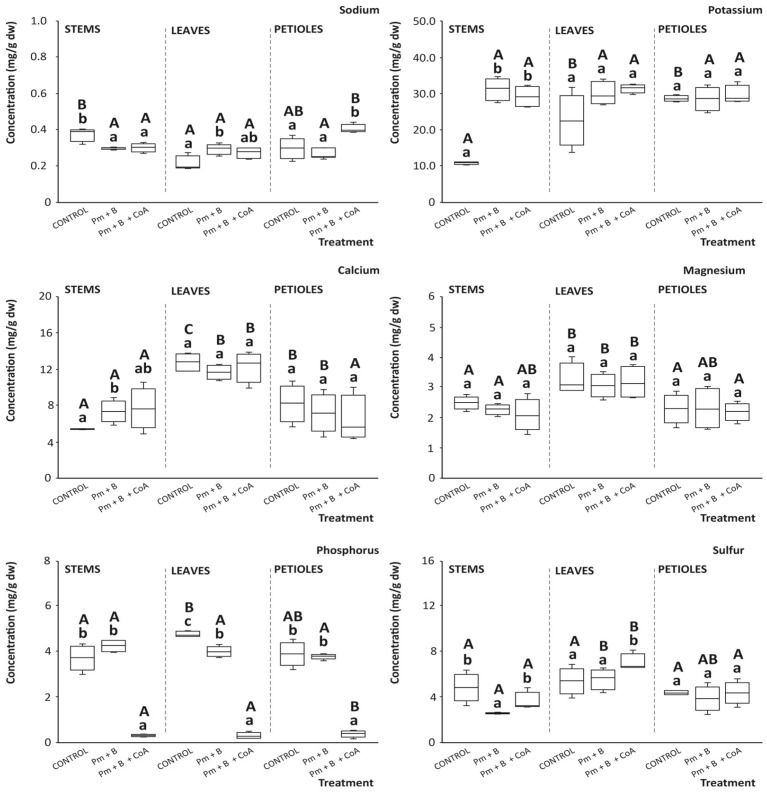
Content of the mineral nutrients sodium, potassium, calcium, magnesium, phosphorus, and sulphur (mg/g dw) in *Moringa oleifera* L. tissues (stems, leaves, and petioles). Boxes with distinct lowercase letters within each separate blot are significantly different between treatments (control, poultry manure + biochar (Pm + B), and poultry manure + biochar + coenzyme A (Pm + B + CoA)), and distinct capital letters within each separate blot are significantly different between tissue, at *p* < 0.05, according to one-way analysis of variance (ANOVA) and Tukey’s multiple range test (*n* = 9).

The mineral contents of stems, leaves, and petioles revealed similar contents in all tissues. However, leaves exhibited significantly higher concentrations of *K* and *Ca*, regardless of the growing condition considered (control, Pm + B, or Pm + B + CoA) (Figure 8). In this regard, it was noticed that the effect of the soil amendments assayed varied depending on the mineral and tissue considered.

Concerning *Na*, the highest concentration under control conditions corresponded to stems (0.4 mg/g dw), which surpassed the level exhibited by leaves and petioles by 20.0%, on average (Figure 8). However, the soil amendments assayed caused different effects, decreasing the concentration in stems and augmenting that in petioles. These different concentrations and changes following the specific agronomical management under study suggest the key influence of the physiological and histological features of the three tissues considered [45].

Generally, the mineral uptake of plants is affected by the soil temperature, which thereby stimulates changes in the architecture and physiology of the rooting system. This is because the surrounding temperature influences the interactions between shoots and roots, thus affecting the pattern of photosynthate translocations of available minerals to other parts of the plant [46]. In addition, concerning the agronomical practices assayed on the soil structure, Zheng et al. (2018) stated that the application of biochar will increase the soil organic matter, nutrients, and cation exchange capacity, as well as replace sodium from exchange sites by introducing *Ca* in soil solution, thereby hindering the availability of *Na* and additional ions to be used by plants [47]. Additionally, the presence of biochar facilitates salt leaching, which consequently reduces the concentration of the available salt to a level suitable for plant growth [48]. Similar trends were observed by Feng et al. (2018), who described that biochar application significantly decreased *Na* ion accumulation in parts of the rice plants [49].

When analysing the concentration of *K*, the main results indicated that trees grown in Pm + B + CoA-amended soil obtained the highest content (31.4 mg/g dw) that surpassed the concentration in leaves of moringa trees grown under control and Pm + B amendment conditions by 6.5% (Figure 8). The increase in the *K* content of the moringa leaves could be attributed to the rise in pH of soil resulting from the application of biochar, which enhances the uptake of *K* by plants [38]. Similarly, stem recorded the highest *K *mineral concentration on soil amended with Pm + B + CoA and Pm + B, respectively, while the control conditions displayed the lowest concentration. No significant difference was observed for this mineral nutrient in the petioles (Figure 8). Previous plant nutrition studies by Tindall et al. indicated that the optimal temperature for uptake of the majority of mineral nutrients is 25 °C [6].

Concerning *Ca*, the assessment of different materials from moringa trees exposed to the range of soil amendments analysed evidenced the highest content in leaves and petioles (28.4 mg/g dw), 16.4% higher than that recorded in stems. The latter was the only tissue that experienced a significant variation in the *Ca* concentration by the soil amendments practised, raising the content in this mineral nutrient by 63.9% (Figure 8). These results are in agreement with previous reports by White and Broadley, who described the capacity of organic soil amendments to enhance *Ca* availability to plants [50]. Furthermore, the rise in *Ca* content in soil applied with Pm + B + CoA could be due to a sufficient level of biologically available nutrients in soils provided by the manure and charcoal dust combination. In this concern, Tonetto de Freitas et al. demonstrated that the slightly lower air temperature and higher relative humidity provide a greater plant water potential, which is associated with a lower pressure in the xylem vessels, possibly favouring water and *Ca *movement throughout the plant tissues, thus contributing to the high *Ca *content found in leaves [51].

The evaluation of the *Mg* content on tissue showed that the level varied significantly through the different agronomical conditions, evidencing the following average decreasing values: leaves (2.9 mg/g dw) > petioles (2.5 mg/g dw) > stems (2.3 mg/g dw) (Figure 8). Thereby, a valuable concentration of this micronutrient was consistent with all agronomic soil characteristics assessed. This is very relevant because Mg is a cofactor of more than 300 enzymes, involved in numerous metabolic cycles, including those participating in the transport of proteins, lipids, carbohydrates, and electrolytes across the cell membrane [52]. Hence, although the application of poultry manure has been reported as a feasible agronomic intervention to improve soil organic matter, as well as the status and quantities of macro-nutrients and micro-nutrients [53,54], in the present work, the effect on the *Mg* level in tissues of the moringa tree was limited.

Regarding *P*, this mineral plays a vital role in metabolic processes, and the levels recorded in the separate moringa tissues assessed help in the production of ATP by plants [6,55]. Indeed, the results of this study indicated that the soil amendments performed did not significantly enhance the *P* status in any of the tissues assessed. Indeed, Pm + B provided values not different from the control in most cases. On the contrary, leaves, stems, and petioles from trees grown in soils after application of Pm + B + CoA displayed the lowest content (Figure 8). This decrease could be associated with the cofactor application, although further research would be required to confirm this linkage.

Finally, the value of the separate moringa tissues as sources of *S* evidenced the following decreasing order of concentration: leaves (6.0 mg/g dw) > petioles (4.2 mg/g dw) > stems (3.7 mg/g dw). In addition, the treatments applied to soils did not provide a clear advantage, except for leaf tissue that, when applying Pm + B + CoA, augmented the *S* concentration by 23.2% (Figure 8). This increase may be due to improved accessibility of *S *during early development phases, thus positively impacting chlorophyll production and the ratio between Rubisco (ribulose 1,5-bisphosphate carboxylase/oxygenase) and soluble protein in sesame plants [56].

**Figure 9:**
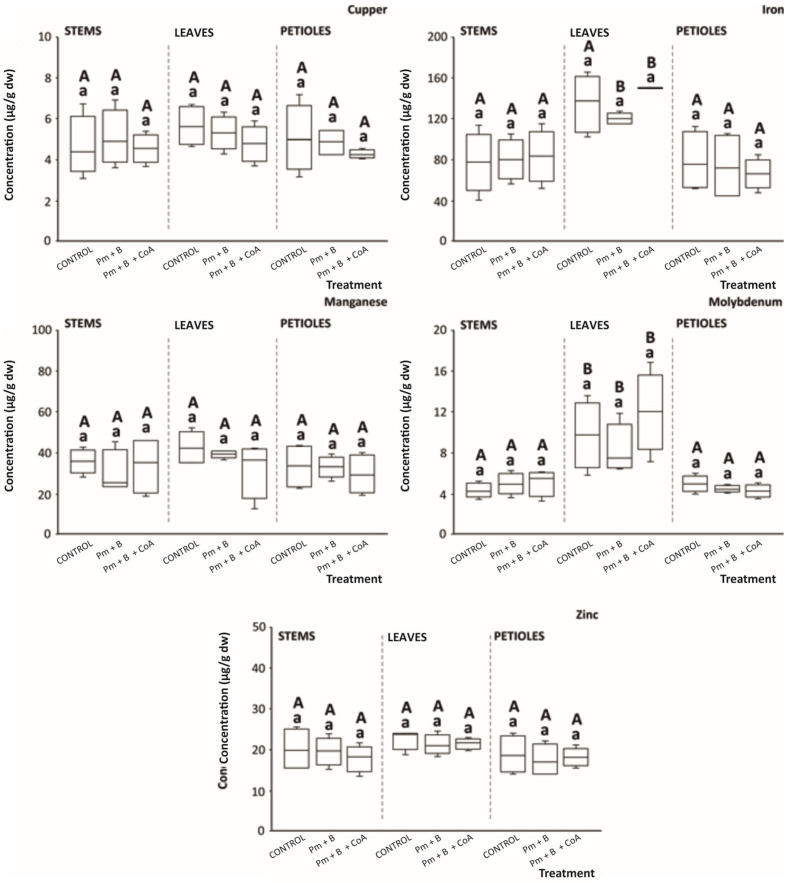
Content of mineral nutrients (μg/g dw) in *Moringa oleifera* L. tissues (stems, leaves, and petioles). Boxes with distinct lowercase letters within each separate blot are significantly different between treatments (control, poultry manure + biochar (Pm + B), and poultry manure + biochar + coenzyme A (Pm + B + CoA)), and distinct capital letters within each separate blot are significantly different between tissues, at *p* < 0.05, according to one-way analysis of variance (ANOVA) and Tukey’s multiple range test (*n* = 9).

Beyond mineral nutrients, trace elements like *Cu*, *Fe*, *Mn*, *Mo*, and *Zn* are required in lesser amounts than the former to obtain high-quality crop yields because of their role in improving plant growth and stress tolerance [55]. In this concern, various elements participate in plant metabolic activities as constituents of enzymes, namely *Zn* and *Cu* in superoxide dismutases or *Mo* in nitrate reductase [57,58]. The evaluation of moringa stems, leaves, and petioles on the content of *Cu*, *Fe*, *Mn*, *Mo*, and *Zn* demonstrated no significant effects of applying Pm + B or Pm + B + CoA towards significantly improving their concentration (Figure 9).

Despite the lack of significant effect of the soil amendments developed in the present work to enhance the profile trace minerals in moringa, the range of concentrations recorded for the separate tissues monitored for *Cu*, *Fe*, *Mn*, *Mo*, and *Zn* (4.29–5.70, 66.84–149.15, 1.62–14.12, 0.65–3.95, and 1.34–5.23 μg/g dw, respectively) agrees with previous assessments [59]. These concentrations have been promoted as significant for mineral content for both plant physiology (e.g., redox balance, plant growth and metabolism, cell respiration, or photosynthesis, among others) and nutritional status (as cofactors of several cell processes in humans, namely oxidative stress prevention or normal functions of the immune system) of the plant material [60,61].

## 4. Results

The present study demonstrated that the integrated use of poultry manure, biochar, and soil cofactors as amendments significantly enhanced the growth performance, biomass yield, and nutritional quality of *M. oleifera* cultivated under field conditions compared to control moringa trees grown without conditioners. In this concern, the main advantages were associated with plant height, stem diameter, number of branches, and leaf production across multiple harvests. Moreover, the analysis of phytochemical composition revealed that moringa leaves, followed by petioles and stems, contained the highest concentrations of bioactive compounds, which also corresponded to plants treated with poultry manure, biochar, and CoA, underscoring the influence of this combination on secondary metabolism, particularly regarding the (poly)phenolic burden. The combined use of poultry manure, biochar, and soil cofactors effectively enhanced *Moringa oleifera* growth, yield, and nutritional quality, offering not only a sustainable, low-cost alternative to synthetic fertilisers for smallholder and degraded farming systems but also a high-quality moringa ingredient for the food and nutraceutical industries rich in health-promoting compounds. These combined outcomes allow linking ecological farming practices with economic and nutritional benefits. These findings point out the soil amendment assessed as particularly relevant to the nutritional scope of regions such as sub-Saharan Africa, including Ghana, where socioeconomic constraints limit the farmers’ access to high-cost agronomic inputs. Hence, the design of interventions based on locally available organic resources (e.g., poultry manure and biochar) will provide a practical and sustainable strategy to improve crop productivity and nutritional quality, particularly for *Moringa oleifera*.

Nevertheless, despite these advantages, the present study has certain limitations since it has been conducted under a single agroecological condition, which may restrict the generalisation of results to other soil types or agroclimatic zones. Additionally, the long-term effects of continuous amendment application on soil health and nutrient dynamics were not assessed. Future research should therefore address these gaps and the economic feasibility across different environments to validate and optimise the management strategy uncovered in the present work.

## Data Availability

The original contributions presented in this study are included in the article. Further inquiries can be directed to the corresponding authors.
